# The Role and Mechanism of Pyroptosis and Potential Therapeutic Targets in Sepsis: A Review

**DOI:** 10.3389/fimmu.2021.711939

**Published:** 2021-07-07

**Authors:** Xiangtao Zheng, Weiwei Chen, Fangchen Gong, Ying Chen, Erzhen Chen

**Affiliations:** Department of Emergency, Ruijin Hospital, Shanghai Jiaotong University School of Medicine, Shanghai, China

**Keywords:** sepsis, pyroptosis, caspase, canonical pyroptosis pathway, non-canonical pyroptosis pathway

## Abstract

Sepsis is defined as life-threatening organ dysfunction caused by a dysregulated host response to infection. Recently was been found that pyroptosis is a unique form of proinflammatory programmed death, that is different from apoptosis. A growing number of studies have investigated pyroptosis and its relationship with sepsis, including the mechanisms, role, and relevant targets of pyroptosis in sepsis. While moderate pyroptosis in sepsis can control pathogen infection, excessive pyroptosis can lead to a dysregulated host immune response and even organ dysfunction. This review provides an overview of the mechanisms and potential therapeutic targets underlying pyroptosis in sepsis identified in recent decades, looking forward to the future direction of treatment for sepsis.

## Introduction

Sepsis is life-threatening organ dysfunction caused by a dysregulated host response to infection (1). Epidemiological studies reported a sepsis mortality rate up to 20.6% (2, 3); if patients develop septic shock, the mortality rate could rise to 40%-50% (4). Since there are few effective treatments for sepsis, it is critical to find new therapeutic targets. In the early stage of sepsis, the host activates an immune defense reaction that induces the programmed death of immune cells. As a mode of programmed cell death, pyroptosis participates in the innate immune response, inhibits intracellular pathogen replications, and activates immune cells to phagocytize and kill pathogens (5, 6). Once pyroptosis is out of control, inflammatory reactions are activated in adjacent cells and tissues, which further aggravates the inflammatory injury, leading to a systemic inflammatory reaction, and eventually causing organ failure or septic shock (7–10). This review primarily focuses on the progress of potential therapeutic targets for pyroptosis in sepsis.

## The Development of Pyroptosis

The phenomenon of pyroptosis was first described by Sansonettiin et al. in 1992; they reported that the death of macrophages infected with *Shigella flexneri* was caspase-1 dependent rather than the traditional form of caspase-3 dependent cell death (11). In 1998, Hersh et al. found that caspase-1 played an important role in this specific mode of cell death. The authors reported that *S. flexneri* could not induce death in caspase-1-knockout macrophages (12). In 2001, Brennan and Cookson discovered a similar phenomenon in macrophages infected by Salmonella typhimurium and named this form of programmed cell death “Pyroptosis” (7, 13). In 2008, Fink et al. found that during pyroptosis, DNA became fragmented and the cell membrane was damaged, which caused intracellular content release, inducing a serious inflammatory reaction (14). In 2011, Kayagaki and colleagues discovered that caspase-11 could induce the death of mouse macrophages; this process was similar to caspase-1-mediated pyroptosis and was termed the “non-canonical pyroptosis pathway” (15). However, it is still unknown how caspases activate pyroptosis. Many studies reported that gasdermin D(GSDMD) is the inflammatory caspase substrate. When caspases were cleaved and activated, they could release intracellular substances by forming pores on the cell membrane and eventually induce pyroptosis (16–19). In 2017, Shao’s team discovered that chemotherapy drugs induced cell death by cleaving GSDME through caspase-3 to induce pyroptosis (20). In 2018, it was found that caspase-8 could also cleave GSDMD, thus inducing pyroptosis during *Yersinia* infection (21, 22).

## Pyroptosis Versus Apoptosis

Although apoptosis and pyroptosis are both types of programmed cell death, there are differences in mechanisms, cell morphologies and biological effects. Apoptosis is mediated by apoptosis-related caspases (e.g., Caspase-3/8/9) and ultimately ends in non-inflammatory necrosis. During the process, nuclear condensation, DNA cleavage and apoptotic bodies can be observed in apoptotic cells. Conversely, pyroptosis is mediated by inflammatory caspases (e.g., caspase-1/4/5/11) that induce nuclear condensation and DNA cleavage, as well as pore formation on the cell membrane, resulting in cell swelling and rupture, that destroys cell membrane integrity, causing inflammatory necrosis (23–26).

## The Molecular Mechanism of Pyroptosis

**Figure 1:**
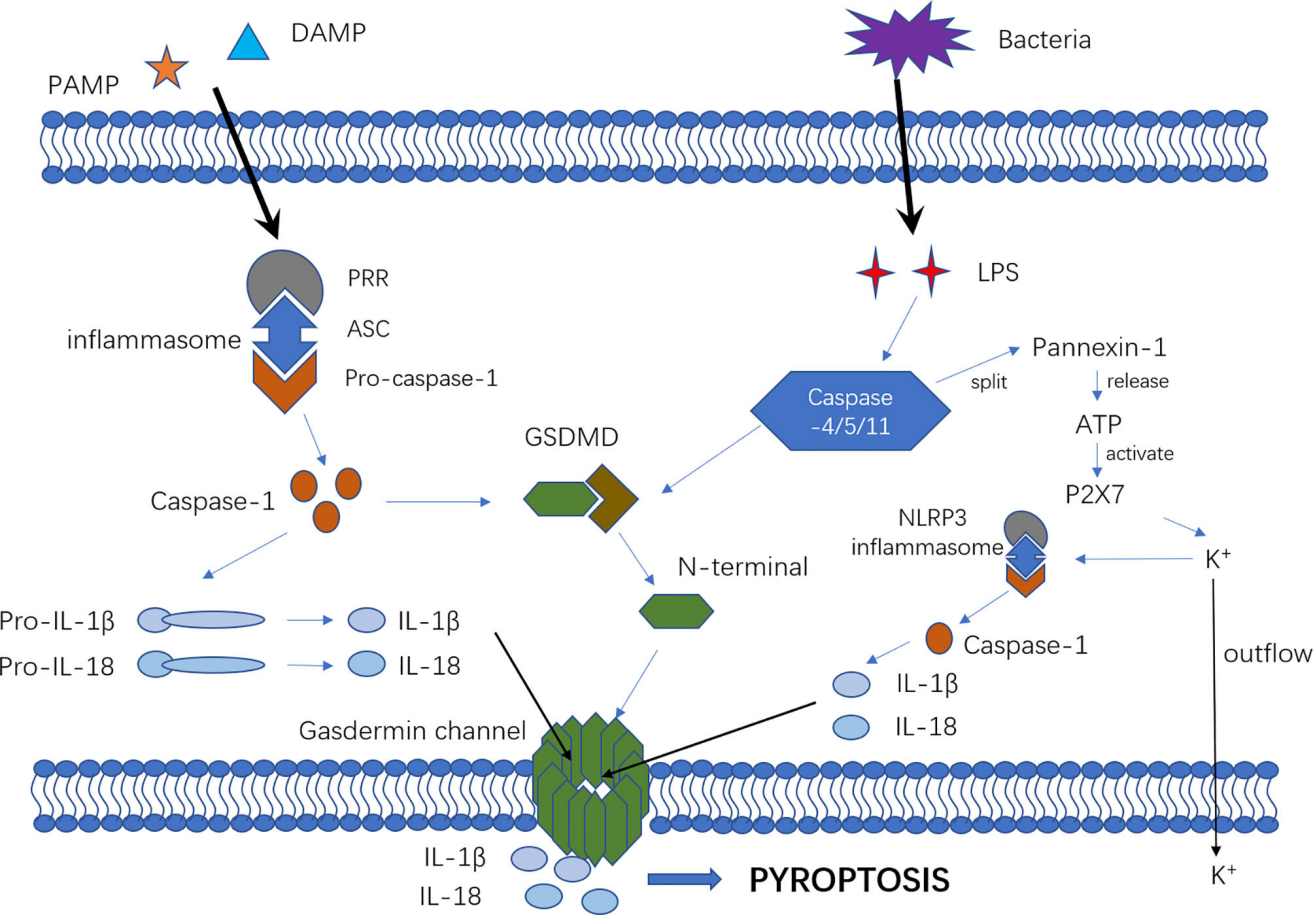
The mechanism of canonical and non-canonical pyroptosis pathway. In the canonical pyroptosis pathway, intracellular pattern recognition receptors (e.g., NLRP1B, NLRP3, NLRC4, etc.) recognize the stimulus signals of pathogens and bind to pro-caspase-1 through the adaptor protein ASC to form a multi-protein complex that can activate caspase-1 protein. In the non-canonical pyroptosis pathway, intracellular LPS directly binds and activates caspase-11/4/5 protein to initiate pyroptosis. After inflammatory caspase activation, pro-IL-1β and pro-IL-18 are cleaved to active IL-1β and IL-18. The portion of GSDMD that connects the N- and C-terminals is rapidly cleaved to remove the inhibitory effect of the C-terminal on the N-terminal. Then, the N-terminal of GSDMD connects with phosphatidylinositol(PI) on the cell membrane, resulting in an oligomerization effect and formation of the “gasdermin channel”. Ion movement through this channel destroys the osmotic balance, leading to cell swelling, membrane dissolution, cell content release, and inflammatory response. Furthermore, cytosolic LPS stimulation induced caspase-11-dependent cleavage of the pannexin-1 channel followed by ATP release, which in turn activated the P2X7 receptor to cause ATP-induced loss of intracellular K+, NLRP3 inflammasome activation and IL-1b secretion.

Pyroptosis mechanisms generally include the caspase-1-dependent pathway (canonical pathway) and caspase-1-independent pathway (non-canonical pathway) that is induced by human caspase-4/5 or mouse caspase-11 (**Figure 1**).

In the canonical pyroptosis pathway, intracellular pattern recognition receptors(PRRs) including Nod-like receptor (NLR) family pyrin domain containing 3(NLRP3), NLR family caspase activation and recruitment domain (CARD) containing 4(NLRC4) and NLR family pyrin domain -containing 1B(NLRP1B) recognize pathogenic stimuli and bind to pro-caspase-1 through the adaptor protein apoptosis-associated speck-like protein contain a CARD(ASC) (27–29) to form a multi-protein complex that can activate caspase-1 protein. In the non-canonical pyroptosis pathway, intracellular lipopolysaccharide (LPS), an activator of non-canonical inflammasomes (30), directly binds and activates caspase-11/4/5 protein to initiate pyroptosis (15). After inflammatory caspases are activated, pro-interleukin-1β (pro-IL-1β) and pro-IL-18 are cleaved to active IL-1β and IL-18, which are released extracellularly to recruit inflammatory cells and enhance the inflammatory response. The connection between the N-terminal and C-terminal of GSDMD is rapidly cleaved to remove the inhibitory effect of the C-terminal on the N-terminal, which then connects with the phosphatidylinositol(PI) on the cell membrane, promoting an oligomerization effect and formation of the “gasdermin channel” (16–18, 31). The formation of numerous micropores on the cell membrane can destroy the osmotic balance, leading to cell swelling and membrane dissolution, followed by the release of cell contents and exacerbating the inflammatory response (16, 24, 32). Furthermore, cytosolic LPS stimulation induces caspase-11-dependent cleavage of the pannexin-1 channel and subsequent ATP release, which in turn activates the P2X7 receptor to cause ATP-induced loss of intracellular K^+^, NLRP3 inflammasome activation and IL-1b secretion. Therefore, NLRP3 might be a vital bridge between the canonical and non-canonical pyroptosis pathways (33–36).

**Figure 2:**
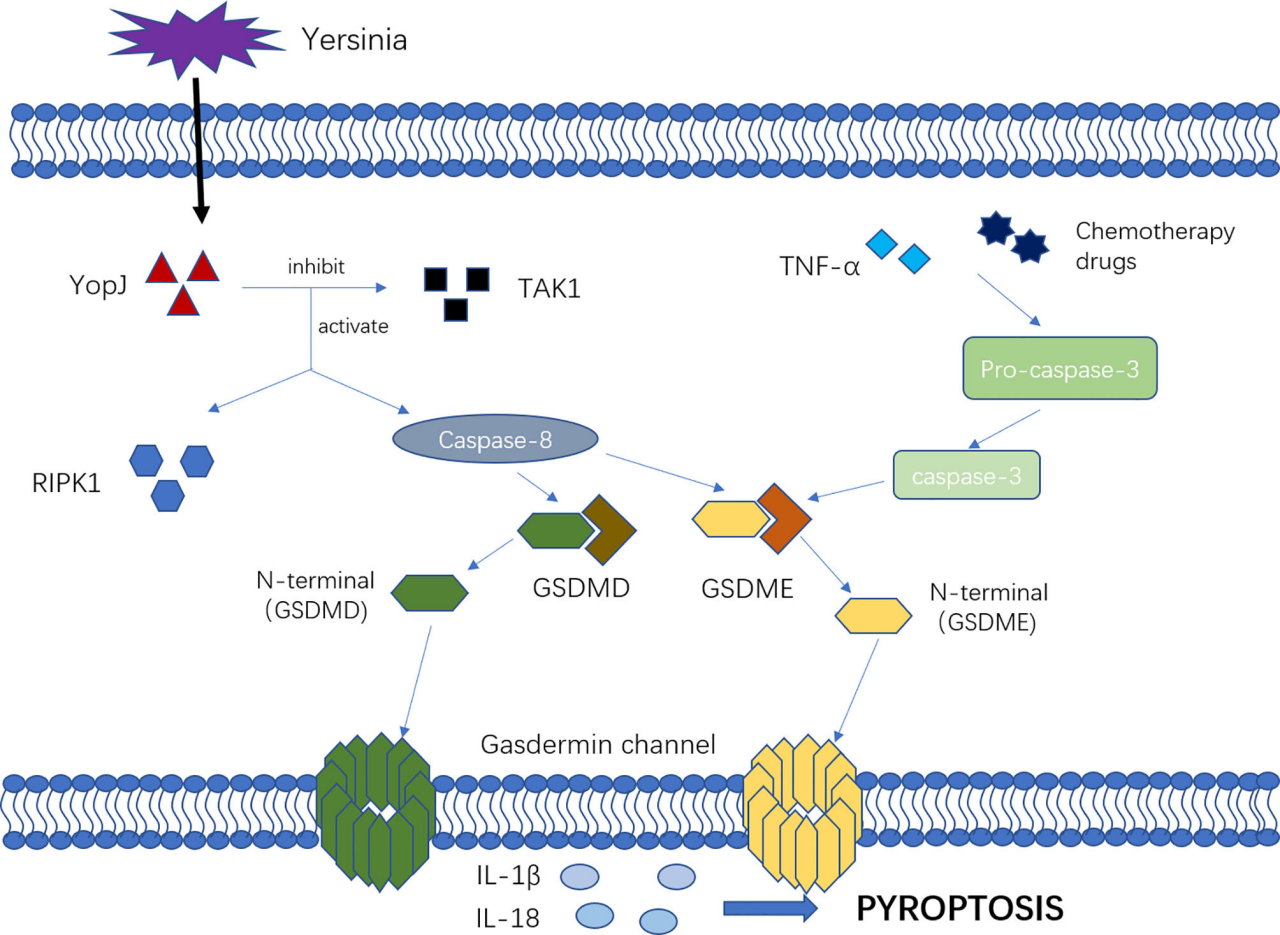
The mechanism of new pyroptosis pathways. Activated by TNF-α or chemotherapy drugs, Caspase-3 cleavage and activate GSDME, releasing N-terminal binding to the cell membrane, forming “gasdermin channel” and inducing cell pyroptosis. Additionally, the pathogenic Yersinia could inhibit TAK1 *via the* effector YopJ, and then activated RIPK1 as well as Caspase-8, which would cleave GSDMD and GSDME, forming the “gasdermin channel” on the cell membrane, mediating pyroptosis and leading to the inflammatory response.

There are new mechanisms of pyroptosis mediated by caspase-3 and caspase-8 (**Figure 2**). Caspase-3 was previously considered as a marker and key molecule of cell apoptosis, but many studies showed that it was also involved in the process of pyroptosis. Activated by tumor necrosis factor-α (TNF-α) or chemotherapy drugs, caspase-3 can specifically cleave and activate gasdermin E (GSDME), releasing the N-terminal to bind to the cell membrane, forming the “gasdermin channel” and inducing cell pyroptosis. In the setting of high GSDME expression, activated caspase-3 can induce pyroptosis, but low GSDME expression induces apoptosis (37, 38). Other studies have shown that caspase-8 is involved in pyroptosis. Sarhan and colleagues found that pathogenic *Yersinia* could inhibit transforming growth factor β-activated kinase 1 (TAK1) *via* the effector YopJ, and then activated receptor-interacting protein kinase 1 (RIPK1) and Caspase-8. Next, caspase-8 cleaves GSDMD and GSDME, forming the “gasdermin channel” on the cell membrane that mediates pyroptosis and lead to the inflammatory response (21, 22, 39).

## The Relationship Between Pyroptosis and Sepsis

### Pyroptosis Participates in Sepsis Regulation

Sepsis is life-threatening organ dysfunction caused by a dysregulated host response to infection. It is a global public health problem because of its high fatality, disability rates, and high disease burden.

In the process of sepsis, moderate immune-inflammatory response can effectively defend against pathogens, although they also cause some damage to the tissue. However excessive proinflammatory response or immunosuppression can lead to organ dysfunction or secondary infection (40). Pyroptosis was originally considered as a pathogenic mechanism that damaged host cells, but moderate pyroptosis is actually a host defense mechanism that is conducive to eliminating intracellular pathogens. Both caspase-1-mediated canonical pyroptosis and caspase-4/5/11-mediated non- canonical pyroptosis are involved in clearing intracellular pathogens; these pathways destroying the pathogen’s living environment by reducing the cytosolic compartment, inhibiting the growth and replication of intracellular pathogens, and speeding up pathogen excretion (5, 41–45). As a result, the escaped pathogens are recognized and eliminated by immunocytes. One study showed that activated GSDMD could also induce bacterial cell membranes to form the “gasdermin channel”, which can kill *Escherichia coli*, *Staphylococcus aureus*, and *Listeria monocytogenes* (32). It follows that moderate pyroptosis plays a protective role in the early stage of infection, although it may cause some tissue damage. Conversely, excessive pyroptosis will cause an uncontrolled inflammatory reaction, which greatly accelerates sepsis occurrence and development that contributes to a poor prognosis (8, 46–48).

Recent studies into the mechanism of pyroptosis have clarified the relationship between sepsis and pyroptosis. Duo et al. found that miR-21, an important positive regulator of pyroptosis and septic shock, regulated the nuclear factor kappa B(NF-κB) pathway and NLRP3-mediated pyroptosis through protein A20 (49). The author demonstrated that miR-21 knockdown would inhibit ASC to recruit inflammatory caspases, thereby inhibiting caspase-1 activation and GSDMD cleavage, ultimately inhibiting LPS-induced pyroptosis and septic shock. Another study showed that caspase-11 activated by LPS could induce pyroptosis and severe inflammatory response through pannexin-1 and P2X7 signaling, which may be a potential target to treat Gram-negative bacteria sepsis (33). Song et al. found that sphingosine-1-phosphate receptor 2 was a potential therapeutic target for sepsis since its knockout could reduce caspase-11 activity in macrophage, inhibit pyroptosis and alleviate sepsis (50). Deng et al. demonstrated that hepatocyte-released high mobility group box 1 (HMGB1) played a significant part in the development of endotoxemia and bacterial sepsis, which could mediate caspase-11-dependent pyroptosis and sepsis lethality by delivering extracellular LPS into the cytosol of macrophages and endothelial cells, where LPS activates caspase-11 (51). It was also shown that binding of transmembrane protein 173 (TMEM173) to the inositol-1, 4, 5-triphosphate type I receptor could control calcium release in the endoplasmic reticulum of macrophages and monocytes. Increased cytoplasmic calcium levels could drive GSDMD cleavage and activation, triggering the release of tissue factors that are key promoters of blood coagulation. Additionally, Inhibiting the TMEM173 pathway was shown to correct diffuse intravascular coagulation (DIC), prevent multiple organ failure, and improve the survival rate of septic animals (52).

### Pyroptosis Mediates Sepsis-Related Organ Damage

The molecular mechanisms and potential therapeutic targets of sepsis-related organ damage have gradually been explored.

A study of sepsis-associated DIC showed that bacterial endotoxin activated tissue factors of the exogenous coagulation pathway are stimulated by caspase-11, which activates the coagulation cascade by triggering “gasdermin channel” formation and exposure to phosphatidylserine (53). Additionally, platelet endothelial cell adhesion molecule-1 could protect against sepsis-associated DIC by inhibiting macrophage pyroptosis; this pathway also plays an important role in restoring the vascular permeability barrier following inflammatory stimulation (54).

With regard to sepsis-associated acute kidney injury (AKI), activation of caspy2, a zebrafish inflammatory caspase homologous to caspase-4/5/11, could specifically cleave gasdermin Eb (GSDMEB), releasing its N-terminal and mediating pyroptosis in mammals. The authors reported that the caspy2-GSDMEb signaling pathway was essential in LPS-induced fatal renal tubular injury caused by septic shock, and the specific GSDMEB inhibitor Ac-FEID-CMK could reduce the incidence of septic AKI and zebrafish mortality (55). These results highlight potential therapeutic targets of septic AKI. Notably, caspase-11 gene knockout reduces the expression of pyroptosis-related proteins of renal tubular epithelial cells (RTECs), suggesting that caspase-11 targeting or inhibition of RTECs may become a new therapeutic target for septic AKI (56).

Concerning sepsis-associated acute lung injury (ALI), endothelial inflammatory caspase may be an important therapeutic target. Caspase-11 knockout was shown to reduce endotoxemia-induced neutrophil aggregation, pulmonary edema, and death (57). In the cecal ligation and puncture (CLP)-induced ALI model, inhibition of HMGB1 expression could reduce caspase-11-dependent pyroptosis in lung tissue, thereby ameliorating lung injury (58). Additionally, phospholipid scramblase4 (PLSCR4) could transport phospholipids outside the membrane, thereby blocking the N-terminal of GSDMD forming the “gasdermin channel”, inhibiting pyroptosis and alleviating lung injury (59).

Studies of sepsis-related cardiac dysfunction have shown that CXADR-like membrane protein (CLMP) was involved in myocardial cell pyroptosis after myocardial infarction in mice, but knockdown of CLMP expression enhanced myocardial cell pyroptosis and aggravated myocardial injury (60). In addition, binding of TMEM173 (also known as stimulator of interferon gene) with type-I interferons regulatory factor 3 under LPS stimulation could increase NLRP3 expression, promote pyroptosis, and aggravate sepsis mediated myocardial injury (61). Some *in vitro* and *in vivo* experiments showed that zinc finger antisense 1 (ZFAS1), as the long noncoding RNA of miR-590-3p regulating AMP-activated protein kinase/mammalian target of rapamycin (AMPK/mTOR) signal transduction under induction of the transcription factor SP1, could induce cardiomyocyte pyroptosis and aggravate sepsis-induced cardiac dysfunction (62). These findings indicate the ZFAS1/miR-590-3p/AMPK/mTOR regulatory network may provide a new therapeutic direction for the development of drugs for sepsis-induced cardiac dysfunction.

### Application of the Therapeutic Targets of Pyroptosis in Sepsis

In addition to the sepsis treatment targets mentioned above, possible applications of therapeutic targets of pyroptosis have been highlighted to explore their therapeutic effect and application prospects in sepsis.

#### Caspase-Related Inhibitors

Braun et al. (63) and Hotchkiss et al. (64) initially reported that the broad-spectrum caspase inhibitor Z-VAD-FMK had a protective effect against pneumococcal meningitis in a New Zealand white rabbit model and sepsis mouse model, respectively. Subsequent studies found that Z-VAD-FMK could significantly reduce IL-1β release in patients with LPS-and *S. aureus*-induced sepsis by inhibiting caspase activity (65). Moreover, the broad-spectrum caspase inhibitor VX-166 exhibited strong anti-apoptotic and anti-inflammatory effects by inhibiting IL-1β and IL-18 release in the CLP rat model and showed significant therapeutic effects against sepsis (66).

An increasing number of researchers are focusing on specific caspase inhibitors rather than broad-spectrum caspase inhibitors because it was difficult to clarify which caspases were contributing to the observed effects. One study reported that the caspase-1 inhibitor VX-765 reduced caspase-1 expression in the brain tissue of the sepsis mouse model and reduce the production of mature IL-1β, which inhibited pyroptosis and eventually attenuated the inflammatory response (67). Chen et al. found that nitrosonisoldipine, a photodegradation product of the calcium channel inhibitor nisoldipine, was a selective inhibitor of inflammatory caspases, that could inhibit noncanonical pyroptosis and protect against pyroptosis (68). In conclusion, caspases are key molecules of cell pyroptosis that can be significantly repressed by its specific inhibitors, thereby hampering pyroptosis and reducing damage caused by infectious diseases. Although specific caspase inhibitors may be a potential treatment for sepsis, but they have not yet been used in clinical practice, and more research is needed to convert promising experimental results into effective clinical drugs.

### Relevant Effective Ingredients of Herbal Medicines and Other Drugs

Recent studies have explored the effects of some components of Chinese herbal medicine on pyroptosis in sepsis. Cinnamon was shown to inhibit the activation of NLRP3, NLRC4, and absent in melanoma 2 (AIM2) inflammasomes, thereby reducing IL-1β secretion and improving the survival rate of LPS-induced septic shock mice (69, 70). Glaucocalyxin A is a bioactive ent-kauranoid diterpenoid derived from herbal medicine that can alleviate LPS-induced septic shock and the inflammatory response by inhibiting NLRP3 inflammasome activation (71). The ginsenoside metabolite protopanaxatriol can play a therapeutic role in inflammatory disorders by inhibiting inflammation-mediated activation of the NLRP3 inflammasome, ASC oligomerization, and IL-1β secretion (72). Myricetin inhibits NLRP3 inflammasome assembly by promoting non-reactive oxygen species (ROS)-dependent NLRP3 ubiquitination and reducing ROS-dependent ASC ubiquitination; this blocks the interaction between ASC and NLRP3, inhibits ASC aggregation, and alleviates the sepsis-induced inflammatory response (73). Additionally, the curcumin analog AI-44 can promote interaction between peroxidase 1 (PRDX1) and pro-caspase-1 by targeting PRDX1. These events hamper the binding of pro-caspase-1 to ASC, inhibiting NLRP3 inflammasome activation and alleviating LPS-induced sepsis injury in mice (74). With further development, it is believed that Chinese herbal medicine will have broader application prospects for the clinical treatment of sepsis in the future.

Chen and colleagues found that L-epinephrine promoted protein kinase A activation by regulating the ADRA2B-ADCY4-PDE8A-PKA axis, thereby blocking caspase-11-mediated IL-1β maturation, GSDMD cleavage, and danger-associated molecular pattern (DAMP) release to restrict inflammasome activation and macrophage pyroptosis (75). Xu et al. reported that estrogen could alleviate liver injury in septic rats by alleviating mitochondrial dysfunction caused by oxidative stress and inhibiting the oxidative stress-mediated pyroptosis signaling pathway (76). And colleagues found that melatonin could inactivate NF-κB induction, reduce NLRP3 expression and alleviate the inflammatory state of sepsis (77). Nagaoka et al. found that the antibacterial peptide LL-37 was beneficial to protect the function of multiple organs by inhibiting the release of macrophage chemotactic factor, promoting the release of neutrophil extracellular traps, and enhancing the inflammatory response to improve the survival rate of sepsis in mice (78). LL-37 may therefore be a useful drug for sepsis because of its antibacterial and anti-pyroptosis abilities. Wang et al. found that carbon monoxide could reduce the mortality of septic rats by inhibiting the cleavage of caspase-1 and caspase-11, thereby reducing the release of IL-18, IL-1β, and HMGB1 and limiting intestinal mucosal permeability and mucosal damage (79). Yang et al. found that prophylactic application of glutamine could promote liver cell pyroptosis to clear pathogens in the early stage of sepsis, but inhibited pyroptosis in the advanced stage of sepsis (80). Wang and colleagues reported that dihydromyricetin could alleviate sepsis-induced acute lung injury by inhibiting NLRP3 inflammasome-dependent pyroptosis in mouse models (81). Hu et al. showed that disulfiram could inhibit pyroptosis and IL-1β release by preventing the N-terminal of GSDMD from forming the “gasdermin channel” on the cell membrane, thus improving the poor prognosis of LPS-induced sepsis in a mouse model (82).

Collectively, the existing data suggest that it is workable to intervene in sepsis and improve patient prognosis by inhibiting cell pyroptosis with drugs or specific compounds. However, there are currently no clinical treatments for this purpose and basic and randomized controlled trials are still needed to demonstrate the efficacy of this approach.

## Conclusion and Prospect

In summary, pyroptosis is an important immune response that plays a significant role in sepsis occurrence and development. In the early stage of sepsis, the organism induces pyroptosis to inhibit intracellular replication of pathogens and accelerate their elimination. If the infection is not controlled, a large number of pathogens will invade the blood and cells to escape identification and elimination by the immune system; during the process, pathogen-associated molecular patterns and DAMPs are released to induce massive pyroptosis, which increases IL-18 and IL–1β levels to aggravating the systematic inflammatory response, eventually leading to organ failure and septic shock. In clinical practice, it is appropriate to induce and inhibit pyroptosis in the early and advanced stage of sepsis, respectively. However, there is still a lack of research on the appropriate timing for intervening in pyroptosis. In recent years, numerous studies have investigated potential therapeutic targets and treatments for sepsis, including broad-spectrum inhibitors, specific caspase inhibitors, and other compounds. All these interventions have shown some therapeutic effects on sepsis models, which brings hope for effective sepsis control. However, research into specific drugs is still lacking, and their effectiveness, targeting, and safety still need further investigation. We believe that it is feasible to regulate sepsis occurrence and development by intervening in pyroptosis, and subsequent research on its mechanism and the development of new drugs will open up new avenues to treat sepsis.

## Author Contributions

All authors participated in the design of the structure of the manuscript. WC and EZC supplied critical suggestions. XZ wrote the manuscript and WC participate in revise the manuscript. All authors contributed to the article and approved the submitted version.

## Funding

This study was funded by the National Natural Science Foundation of China (No. 81772107), Clinical Research Plan of SHDC (No. SHDC2020CR1028B), Shanghai Jiao Tong University School of Medicine (No. DLY201803), Shanghai Science and Technology Innovation Fund (No. 18411950900), and supported by Program for Outstanding Medical Academic Leader to EZC.

## Conflict of Interest

The authors declare that the research was conducted in the absence of any commercial or financial relationships that could be construed as a potential conflict of interest.
